# The neural correlates of texture perception: A systematic review and activation likelihood estimation meta‐analysis of functional magnetic resonance imaging studies

**DOI:** 10.1002/brb3.3264

**Published:** 2023-09-25

**Authors:** Jessica Henderson, Tyler Mari, Danielle Hewitt, Alice Newton‐Fenner, Timo Giesbrecht, Alan Marshall, Andrej Stancak, Nicholas Fallon

**Affiliations:** ^1^ School of Psychology University of Liverpool Liverpool UK; ^2^ Institute of Risk and Uncertainty University of Liverpool Liverpool UK; ^3^ Unilever, Research and Development Port Sunlight UK; ^4^ Department of Electrical Engineering and Electronics University of Liverpool Liverpool UK

**Keywords:** activation likelihood estimation meta‐analysis, discriminative touch, functional magnetic resonance imaging, systematic review

## Abstract

**Introduction:**

Humans use discriminative touch to perceive texture through dynamic interactions with surfaces, activating low‐threshold mechanoreceptors in the skin. It was largely assumed that texture was processed in primary somatosensory regions in the brain; however, imaging studies indicate heterogeneous patterns of brain activity associated with texture processing.

**Methods:**

To address this, we conducted a coordinate‐based activation likelihood estimation meta‐analysis of 13 functional magnetic resonance imaging studies (comprising 15 experiments contributing 228 participants and 275 foci) selected by a systematic review.

**Results:**

Concordant activations for texture perception occurred in the left primary somatosensory and motor regions, with bilateral activations in the secondary somatosensory, posterior insula, and premotor and supplementary motor cortices. We also evaluated differences between studies that compared touch processing to non‐haptic control (e.g., rest or visual control) or those that used haptic control (e.g., shape or orientation perception) to specifically investigate texture encoding. Studies employing a haptic control revealed concordance for texture processing only in the left secondary somatosensory cortex. Contrast analyses demonstrated greater concordance of activations in the left primary somatosensory regions and inferior parietal cortex for studies with a non‐haptic control, compared to experiments accounting for other haptic aspects.

**Conclusion:**

These findings suggest that texture processing may recruit higher order integrative structures, and the secondary somatosensory cortex may play a key role in encoding textural properties. The present study provides unique insight into the neural correlates of texture‐related processing by assessing the influence of non‐textural haptic elements and identifies opportunities for a future research design to understand the neural processing of texture.

## Graphical abstract

Our review provides unique insight into the understanding of texture perception in the brain by investigating texture processing compared to non‐haptic and haptic baselines. Activation likelihood estimation demonstrated concordant activation for texture processing in primary and high‐order sensorimotor regions. Further, contrast analysis suggested texture processing preferentially recruits high‐order integrative structures.

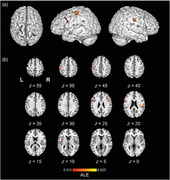

## 1 INTRODUCTION

Humans typically explore and gather haptic information using discriminative touch through the glabrous skin on their hands and digits (Gibson, 1962; Lederman & Klatzky, 1993; Wagner & Gibson, 2016). Previous research investigating texture perception and brain activation commonly focus on one textural feature, with roughness the most studied (Hollins et al., 2000). This meta‐analysis aimed to collate research articles using functional magnetic resonance imaging (fMRI) methods to identify regions of the brain associated with texture perception of various stimuli during discriminative touch. For the purpose of this review, texture perception was defined as the activation of low‐threshold mechanoreceptors (LTMRs) and the dorsal column‐medial lemniscus (DCML) pathway (Gardner & Johnson, 2012a). Importantly, this excludes thermal perception via thermoreceptors and the spinothalamic tract, which has previously been included as a dimension of texture (Okamoto et al., 2013).

The glabrous skin of hands contains LTMRs, which transduce incoming tactile information from surface texture (Gomez‐Ramirez et al., 2016; Harvey et al., 2013; Johnson et al., 2000; McGlone & Reilly, 2010). Tactile information from LTMRs travels through the DCML pathway to the brain, and thalamocortical afferents convey signals to the primary (SI) and secondary somatosensory cortices (SII; Klingner et al., 2016; Raju & Tadi, 2021)). In humans, texture processing elicits bilateral activation in the SI and SII (Genna et al., 2018; Simões‐Franklin et al., 2011). Lesions in the macaque SI and SII lead to impairment of texture perception (Garcha & Ettlinger, 1980; Randolph & Semmes, 1974). Moreover, the SII is hypothesized to be responsible for roughness discrimination (Kitada et al., 2005; Sathian et al., 2011; Servos et al., 2001; Stilla & Sathian, 2008).

The activation of LTMRs requires voluntary movement or dynamic passive touch and contact pressure. The MI and non‐primary motor regions, split into the supplementary motor area (SMA) and premotor cortex, are responsible for planning and initiating limb movements (Rizzolatti & Kalaska, 2012; Rizzolatti & Luppino, 2001). Therefore, in contrast to employing voluntary movement (i.e., active touch), the MI and premotor areas would be activated due to motor preparation and execution.

Somatosensory information is also processed in the insula, with the posterior region of the insular cortex found to be functionally connected to sensorimotor areas including the SI, SII, primary motor cortex (MI), and SMA (Deen et al., 2011; Taylor et al., 2009). The insula is conceptualized as an integration hub as it is connected to many brain regions and is associated not only with sensory inputs (Craig et al., 2000; Jensen et al., 2016; Segerdahl et al., 2015) but also with affective processing (Björnsdotter et al., 2009, 2014; Morrison, 2016; Olausson et al., 2016) and higher level cognition such as decision‐making (Gogolla, 2017; Uddin et al., 2017). Therefore, texture perception in the brain likely involves the insula (Kitada et al., 2005; Stilla & Sathian, 2008).

The posterior parietal cortex (PPC) is linked to multisensory integration, combining inputs from several brain areas, including somatosensory, auditory, visual, motor, cingulate, and prefrontal cortices (Buneo & Andersen, 2006; Whitlock, 2017). Increased activation in the PPC has been demonstrated during grasping, reaching, and interacting with objects (Vingerhoets, 2014). In humans, the PPC can be divided into three regions: the superior parietal lobule (SPL), the inferior parietal lobule (IPL), and the intraparietal sulcus (IPS; Gardner & Johnson, 2012b). The SPL integrates proprioceptive information to guide motor actions (Hadjidimitrakis et al., 2019; Johns, 2014; Kalaska, 1996), while the IPL interprets sensory information to aid in the preparation of motor acts such as hand grasping movement for object manipulation (Bodegård et al., 2001; Vingerhoets, 2014). Similarly, the IPS plays a role in the multisensory processing of vision and touch (Amedi et al., 2005; Cusack, 2005; Grefkes et al., 2002; Peltier et al., 2007; Saito et al., 2003; Tal & Amedi, 2009), contributing to a range of cognitive functions including spatial imagery (Cohen et al., 1996) and attention (Macaluso et al., 2002). Lesions within these areas impact sensorimotor integration and somatosensory processing (Freund, 2001; Murray & Mishkin, 1984); lesions to the SPL and surrounding IPS can impair the sensorimotor integration of proprioceptive signals and cause optic ataxia (Andersen et al., 2014; Perenin & Vighetto, 1988), while lesions to the IPL can cause tactile agnosia (Reed & Caselli, 1994; Reed et al., 1996). Therefore, it is likely that texture discrimination through active touch activates the PPC to aid sensorimotor integration and somatosensory processing.

In the present study, a coordinate‐based meta‐analysis was performed with an activation likelihood estimation (ALE) of published fMRI findings relating to the neural correlates of texture perception (Eickhoff et al., 2009, 2012). First, we aimed to identify key brain regions involved in texture perception at hand and/or digit skin sites using concordance analysis to identify regions of the brain with the highest activation likelihood. Second, we attempted to identify key brain regions involved in texture‐specific perception when controlling for other haptic elements involved in discriminative touch (e.g., location, orientation, and shape). For this purpose, we performed conjunction and contrast analyses to compare fMRI studies which contrasted texture perception with a resting or non‐haptic control with those which used a haptic baseline to control for these non‐texture aspects of discriminative touch.

We hypothesized that areas consistently reported in tactile perception studies would result in activation, which are bilateral SI, SII, and insular cortices. Further, we expected that areas associated with voluntary movement and motor planning would show activation, including bilateral MI, SMA, premotor cortex, and PPC. When controlling for the influence of haptic processing, we anticipated an increased likelihood of activation in medial brain regions associated with higher order processing or texture‐specific processing such as the SII and insular cortex.

## 2 METHOD

This systematic review is reported following the Preferred Reporting Items for Systematic Reviews and Meta‐Analyses guidelines (Moher et al., 2009). The review protocol was registered on Open Science Framework (http://osf.io/kz7mg/?view_only=f47532ac93e64598b56c5c488e651845) on November 3, 2020.

### 2.1 Data search and extraction

Three electronic databases were examined during February 2023 (PubMed, PsycINFO, and Web of Science) using the Medical Subject Headings search terms (magnetic resonance imaging OR fMRI) AND (functional OR brain activation OR neural activity OR BOLD) AND (texture OR rough* OR smooth* OR soft*) AND (touch OR tactile OR haptic OR somatosensory). No date limit was set for the searches. A citation search was conducted of the five most recent research papers accepted for analysis.

### 2.2 Article selection and extraction of data

Article selection consisted of two stages and was conducted by the same two authors (Jessica Henderson and Tyler Mari). First, the title and abstract for all unique search results were assessed separately by the two authors, and studies identified as relevant were retrieved for full‐text review. During the second stage, full‐text articles, retrieved from stage one, were reviewed independently for inclusion, and disagreements were resolved via discussion or presented to a third arbiter (Nicholas Fallon). One author (Jessica Henderson) extracted the relevant coordinate data, which were cross‐checked and confirmed by a second author (Tyler Mari). Studies that reported coordinates in Talairach space were converted into Montreal Neurologic Institute (MNI) using the GingerALE software for analysis and reporting (Eickhoff et al., 2009, 2012; Turkeltaub et al., 2012). Studies that employed a region‐of‐interest (ROI) analysis to investigate the contrast of interest were included in the cohort when whole‐brain statistical data were available from online repositories, such as NeuroVault (Gorgolewski et al., 2015). In such instances, the unthresholded t‐maps resulting from the fMRI analysis were manually thresholded at p<.001 uncorrected voxelwise throughout the whole brain with a p<.05 cluster‐level correction to give whole‐brain results.

### 2.3 Eligibility criteria

**FIGURE 1:**
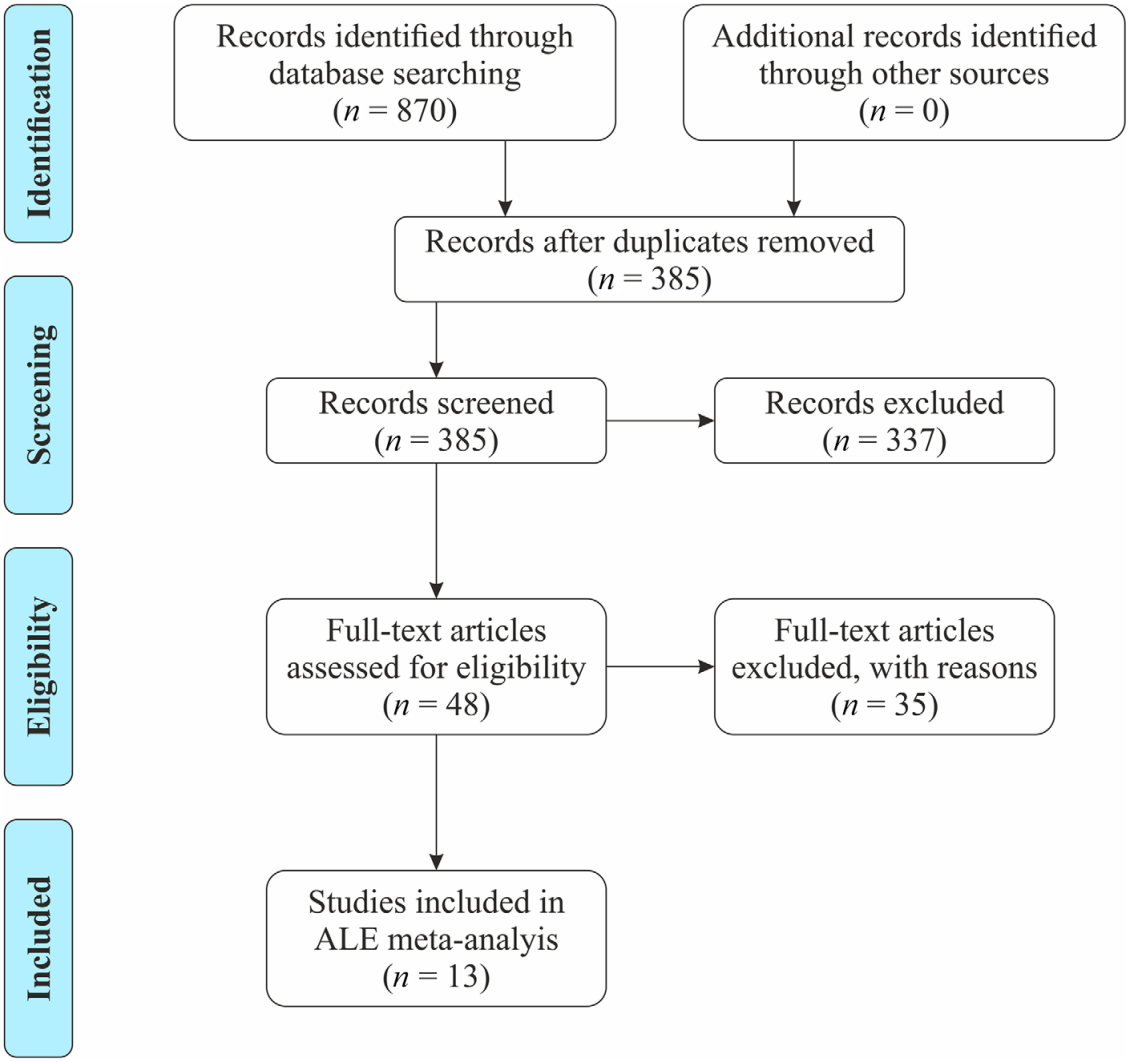
Flow chart depicting the screening process. ALE, activation likelihood estimation.

The criteria for inclusion were as follows: (i) fMRI studies; (ii) original English language articles; (iii) published in a peer‐reviewed journal; (iv) healthy human participants aged 18+; (v) using a paradigm where the hand and/or fingers are either passively or actively stimulated by textured stimuli, that is, three‐dimensional (3D)‐printed texture, natural texture, or man‐made textures; (vi) coordinates were reported in the paper or supplementary material in either MNI (Evans et al., 1994) or Talairach space (Talairach & Tournoux, 1988); (vii) studies which analyzed either of the two contrasts of interest: (1) texture perception through hand and/or finger stimulation compared to non‐haptic control conditions, such as rest, visual control (e.g., visual instructions or rating scales with the absence of textured stimuli) or motor control (e.g., hand motion with the absence of textured stimuli), and (2) texture perception through hand and/or fingers stimulation compared to haptic control conditions, which included shape, location, and orientation tasks. See Figure 1 for a flowchart showing the study selection steps.

ALE meta‐analyses were performed in BrainMap GingerALE v3.0.2 (Eickhoff et al., 2009, 2012; Turkeltaub et al., 2012). The ALE method computes an ALE value for each voxel in the brain and performs tests to determine the null distribution of the ALE statistic at each voxel, with increased ALE values suggestive of more studies reporting activated peaks in specific loci or at neighbouring voxels using a Gaussian distribution. Next, *p*‐values computed from the previous step are used to calculate a thresholded ALE map, and thereafter cluster analysis is performed on the thresholded map.

For the primary analyses, the comparison of texture perception > control, texture perception > non‐haptic control, and texture perception > haptic control was evaluated with permutation analyses performed with 5000 permutations. First, a cluster‐forming threshold of uncorrected p<.001 was applied (Eickhoff et al., 2012), followed by cluster‐level family‐wise error correction (p<.05) as recommended (Eickhoff et al., 2016). For the secondary analyses, the thresholded ALE images from the primary analysis were compared using conjunction and contrast analyses; this was executed by permutation analysis with 10,000 permutations and a cluster‐level false discovery rate threshold of p<.05, with a minimum cluster size of 200 mm^3^ as recommended (Eickhoff et al., 2016), and in line with previous research (Morrison, 2016).

## 3 RESULTS

A total of 870 articles were identified from searches (PubMed, 244; PsycInfo, 362; Web of Science, 264; Figure 1). Of these, 485 articles were removed due to duplication from repeated searches. An additional 337 articles were removed during the titles and abstracts review stage. Studies excluded at this stage included those where it was clear and obvious that no suitable population was reported (106), not an experimental report published in a peer‐reviewed journal (16), did not use fMRI methods (80), not using suitable textured stimuli (130), and not addressing one of the outcomes outlined (5). Following full‐text review, a further 35 articles were removed including those that used an unsuitable contrast (121), did not utilize appropriate textured stimuli (13), did not conduct an fMRI contrast study (5), were not an experimental report journal article (3), only reported ROI analysis and whole brain data were not available (1; see Section 2.2), or which did not report findings in English (1). This resulted in a final cohort of 13 studies for the analyses of texture perception (Table 1), with the age range of participants recruited being 18–47 years. The citation search did not lead to the inclusion of any additional studies. Studies contributing to this ALE stimulated the right hand, with (Kitada et al., 2006) stimulating both hands and combining results. Therefore, concordant activation in the left and right hemispheres correspond to contralateral and ipsilateral activation, respectively.

**TABLE 1:** Studies included in activation likelihood estimation (ALE) meta‐analysis.

Author	Year	Title	N	Mean age (SD)	Description of tactile stimuli	Stimulation site	Type of stimulation	Task used in contrast	Included experiments
Gurtubay‐Antolin et al.	2018	Neural evidence of hierarchical cognitive control during haptic processing: An fMRI study	17	23.4 (1.5)	Six real 3D objects and six textures	Right palm/hand	Active	Congruency	Haptic texture > Haptic shape
Kim et al.	2015	Decoding accuracy in supplementary motor cortex correlates with perceptual sensitivity to tactile roughness	13	25.3 (3.8)	Five grades of aluminum oxide sandpaper	Right index fingertip	Active	Perception	Haptic texture (3 μm) > rest
									Haptic texture (5 μm) > rest
									Haptic texture (9 μm) > rest
									Haptic texture (12 μm) > rest
									Haptic texture (40 μm) > rest
Kitada et al.	2006	Multisensory activation of the intraparietal area when classifying grating orientation: A functional magnetic resonance imaging study	16	22–47^a^ (range)	Nine rectangular gratings with three degrees of roughness	Right and left middle finger	Passive	Estimation	Roughness task > button press
									Roughness task > tactile orientation^b^
Kitada et al.	2005	Tactile estimation of the roughness of gratings yields a graded response in the human brain: An fMRI study	14	23–26^a^ (range)	Linear gratings with three ridge heights	Right middle fingertip	Passive	Perception	No estimation > Rest
Mueller et al.	2019	Neural correlates of top‐down modulation of haptic shape versus roughness perception	21	25.33 (3.44)	3D‐printed cuboids with five levels of shape and roughness	Right thumb and index finger	Active	Comparison	Roughness > Rest
Podrebarac et al.	2014	Are visual texture‐selective areas recruited during haptic texture discrimination?	13	27^a^	Two 3D shapes with two indented texture patterns	Right hand	Active	Comparison	Haptic texture > haptic shape
Sathian et al.	2011	Dual pathways for haptic and visual perception of spatial and texture information	18	20.8^a^	Textiles attached to a piece of cardboard	Right hand	Active	Comparison	Haptic texture > haptic location
Simões‐Franklin et al.	2011	Active and passive touch differentially activate somatosensory cortex in texture perception	16	23.6^a^	Three grades of aluminum oxide sandpaper	Right middle finger	Active and passive	Estimation	Active and passive > control
Stilla and Sathian	2008	Selective visual‐haptic processing of shape and texture	6	22^a^	3D meaningless objects. Textiles attached to a piece of cardboard	Right hand	Active	Comparison	Haptic texture > haptic shape
Tang et al.	2021	Brain activation related to the tactile perception of touching ridged texture using fingers	10	22 (2.3)	Ridged textures with different edge shapes	Right index finger	Passive	Perception	Sharp shape > rest
									Rounded shape > rest
									Flat shape > rest
Wang et al.	2016	Brain discriminative cognition on the perception of touching different fabric using fingers actively	8	28.6^a^	Silk and linen swatches	Right thumb and index finger	Active	Estimation	Linen > rest
									Silk > rest
Yang et al.	2021	Different activation signatures in the primary sensorimotor and higher‐level regions for haptic three‐dimensional curved surface exploration	20	22 (0.63)	3D‐printed surfaces with four levels of raised dot patterns and four types of curvature, plus one flat surface	Right index and middle finger	Active	Estimation	Roughness estimation > hand motion and visual control
									(Roughness estimation—hand motion and visual control) > (curve estimation—hand motion and visual control)^b^
Yang et al.	2017	Brain networks involved in tactile speed classification of moving dot patterns: The effects of speed and dot periodicity	20	21.9 (2.6)	Two surfaces with identical or pseudo‐randomly distributed dot patterns	Right middle fingertip	Passive	Estimation	Speed classification periodic > visual motor control periodic
									Speed classification non‐periodic > visual motor control non‐periodic

Abbreviation: 3D, three‐dimensional.

^a^ Did not report standard deviation; Estimation = Participants estimated textural properties, for example, roughness; Comparison = Presented participants with two stimuli in succession, and participants had to indicate if the stimuli were identical or different; Congruency = Judge whether a touched stimulus corresponded to an expected stimulus which had been presented before stimulus exploration; Perception = Exploration of the textured stimuli with a rest period between trials.

^b^ Not combined as a single experiment because they contribute to different analyses (i.e., texture perception > non‐haptic control or texture perception > haptic control).

### 3.1 Primary analyses

#### 3.1.1 Texture perception > control

**FIGURE 2:**
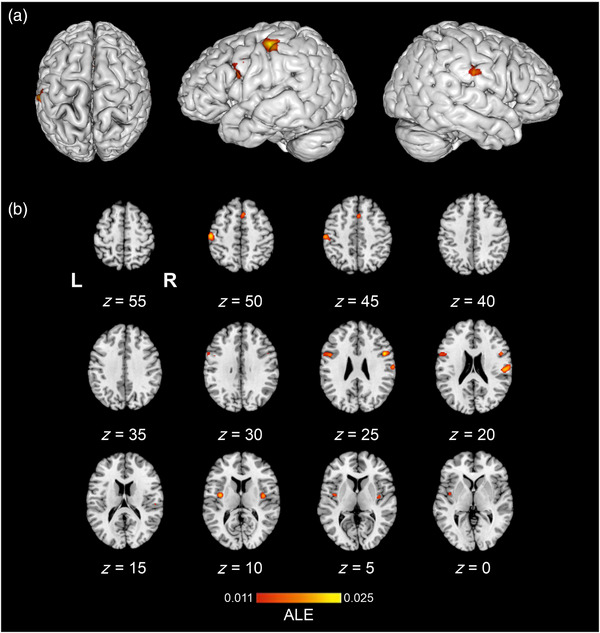
The location of significant activation likelihood estimation (ALE) clusters from the meta‐analysis of concordant activations for texture perception > control. Results are displayed overlaid onto a standardized Montreal Neurologic Institute (MNI) template anatomical brain in (a). Three‐dimensional (3D) surface projection from superior, left, and right views, respectively. (b) As a montage of coronal slices throughout the whole brain, L and R denote the left and right hemispheres, respectively. ALE scores are indicated by the color bar.

Texture perception > control contrast ALE meta‐analysis pooled data from 13 studies which contributed 15 experiments, with a total of 228 participants and 275 reported foci. The results revealed seven significant clusters (Table 2). One cluster originated from the right hemisphere and spanned from the superior temporal gyrus to the postcentral gyrus, corresponding to both the right posterior insula (PI) and SII. One cluster was identified in the left hemisphere from the supramarginal gyrus (SMG) in the IPL to the SI. Further clusters were identified in the bilateral SMA with one peak in the right SMA, two clusters encompassed the bilateral PI, and two clusters encompassed the bilateral ventral premotor cortex (PMv). Significant ALE cluster locations are illustrated in Figure 2.

**TABLE 2:** Locations of significant clusters from the activation likelihood estimation (ALE) map of texture perception > control.

Cluster #	Label	Volume (mm^3^)	BA	*x*	*y*	*z*	#Experiments	ALE
1	Insula R	1752	13	58	−20	20	7	0.021
	Postcentral gyrus R		3	64	−16	22		0.021
2	Postcentral gyrus L	1600	2	−54	−20	48	8	0.023
3	Precentral gyrus L	1424	6	−48	6	24	7	0.019
	Precentral gyrus L		6	−58	8	28		0.014
	Precentral gyrus L		6	−58	2	32		0.012
4	Insula L	1224	13	−36	−6	10	6	0.022
	Insula L		13	−42	−4	2		0.014
5	Inferior frontal gyrus R	984	9	50	8	24	4	0.025
6	Insula R	816	13	40	−8	8	4	0.020
7	Superior frontal gyrus R	816	6	4	16	48	4	0.018

Abbreviations: BA, Brodmann area; L, left hemisphere; R, right hemisphere.

#### 3.1.2 Texture perception > non‐haptic control

**FIGURE 3:**
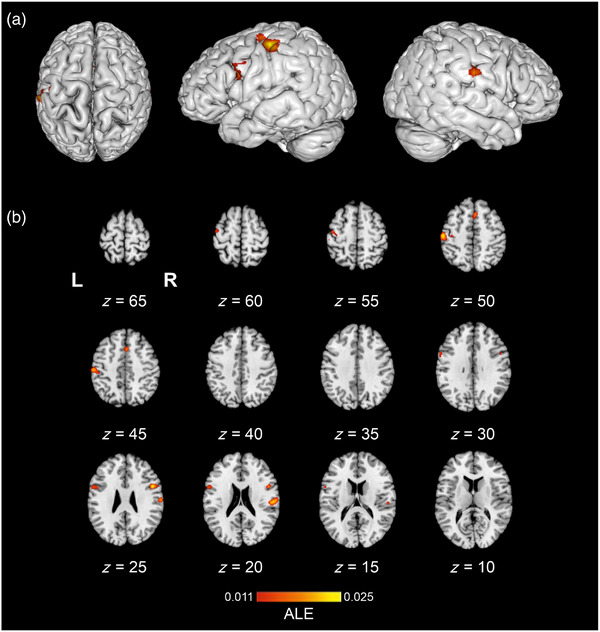
The location of significant activation likelihood estimation (ALE) clusters from the meta‐analysis of concordant activations for texture perception > haptic control. Results are displayed overlaid onto a standardized Montreal Neurologic Institute (MNI) template anatomical brain as a montage of coronal slices throughout the whole brain, L and R denote the left and right hemispheres, respectively. ALE scores are indicated by the color bar.

The texture perception > non‐haptic control (e.g., rest or visual control) contrast ALE meta‐analysis pooled data from nine studies which contributed to nine experiments, with a total of 138 participants and 240 reported foci. The results revealed six significant clusters (Table 3); one cluster spanned from the right superior temporal gyrus to the postcentral gyrus, consistent with both PI and SII. Cluster two, in the left hemisphere, spanned from the SMG in the IPL to the SI. Cluster six corresponded to the left SI and MI. Further clusters encompassed the bilateral SMA and the bilateral PMv. Significant ALE cluster locations are illustrated in Figure 3.

**TABLE 3:** Locations of significant clusters from the activation likelihood estimation (ALE) map of texture perception > non‐haptic control.

Cluster #	Label	Volume (mm^3^)	BA	*x*	*y*	*z*	#Experiments	ALE
1	Insula R	1864	13	58	−20	20	7	0.021
	Postcentral gyrus R		3	64	−16	22		0.021
2	Postcentral gyrus L	1832	2	−54	−20	48	8	0.023
3	Inferior frontal gyrus L	1336	9	−58	6	22	5	0.016
	Precentral gyrus L		6	−58	8	28		0.014
	Precentral gyrus L		6	−58	6	14		0.013
	Precentral gyrus L		6	−58	2	32		0.012
4	Inferior frontal gyrus R	1120	9	50	8	24	4	0.025
5	Superior frontal gyrus R	944	6	4	16	48	4	0.018
6	Postcentral gyrus L	752	3	−44	−12	58		0.016
	Precentral gyrus L		4	−38	−20	52	4	0.013

Abbreviations: BA, Brodmann area; L, left hemisphere; R, right hemisphere.

#### 3.1.3 Texture perception > haptic control

**FIGURE 4:**
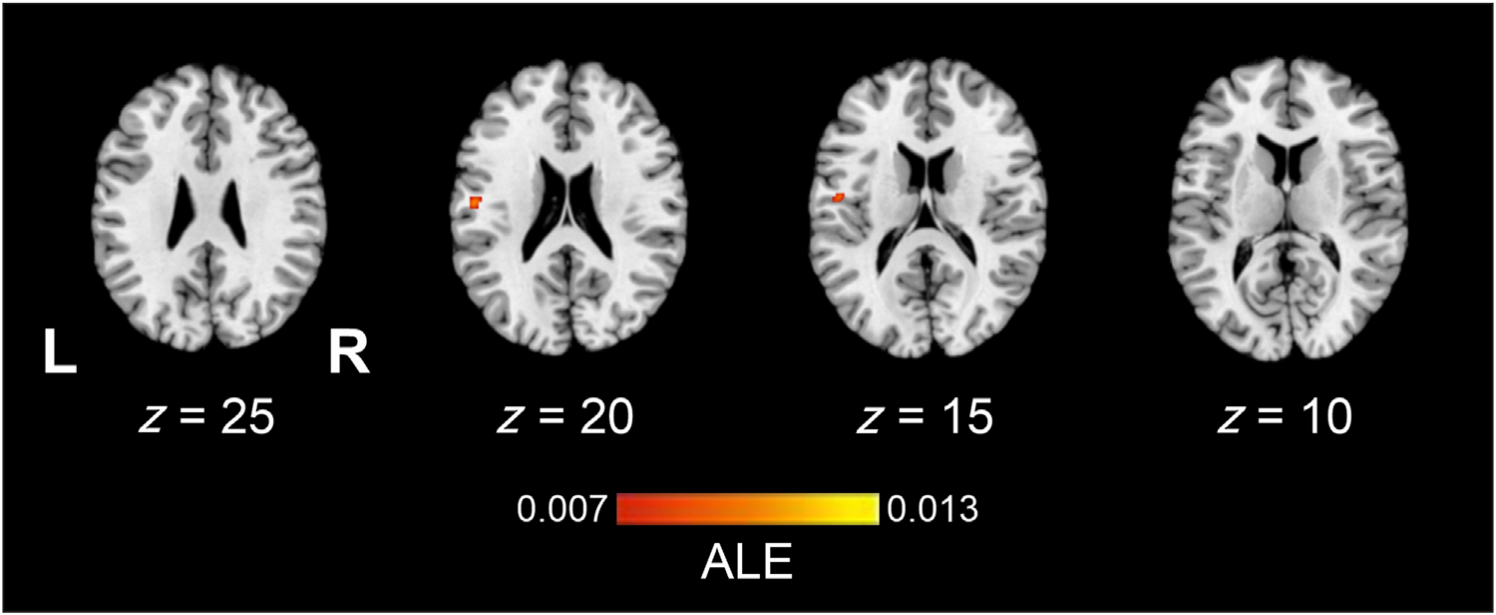
The location of significant clusters from contrast analysis of activation likelihood estimation (ALE) maps for a greater likelihood of texture perception > non‐haptic control relative to texture perception > haptic control. All clusters are overlaid onto a standardized Montreal Neurologic Institute (MNI) template anatomical brain in (a). Three‐dimensional (3D) surface projection from superior, left, and right views, respectively. (b) As a montage of coronal slices throughout the whole brain, L and R denote the left and right hemispheres, respectively. Relative *Z* scores are indicated by the color bar.

The texture > haptic control contrast ALE meta‐analysis pooled data from a total of six studies which contributed to six experiments, with 90 participants and 35 reported foci. Findings demonstrated one significant left hemisphere cluster located in the SII (Table 4). Figure 4 illustrates the location of significant ALE clusters from the meta‐analysis of texture perception when controlling for other haptic processes.

**TABLE 4:** Locations of significant clusters from the activation likelihood estimation (ALE) map of texture perception > haptic control.

Cluster #	Label	Volume (mm^3^)	BA	*x*	*y*	*z*	#Experiments	ALE
1	Postcentral gyrus L	608	43	−54	−14	22	2	0.010
	Postcentral gyrus L		43	−50	−10	16		0.009

Abbreviations: BA, Brodmann area; L, left hemisphere.

### 3.2 Secondary analyses

#### 3.2.1 Conjunction analysis

The conjunction analysis of ALE maps representing texture perception relative to non‐haptic control and texture‐specific perception (relative to haptic control) pooled data from a total of 23 experiments, with a total of 245 participants and 273 reported foci. There were no findings of overlap of activation likelihood coordinates across both contrast types.

#### 3.2.2 Contrast analyses

**FIGURE 5:**
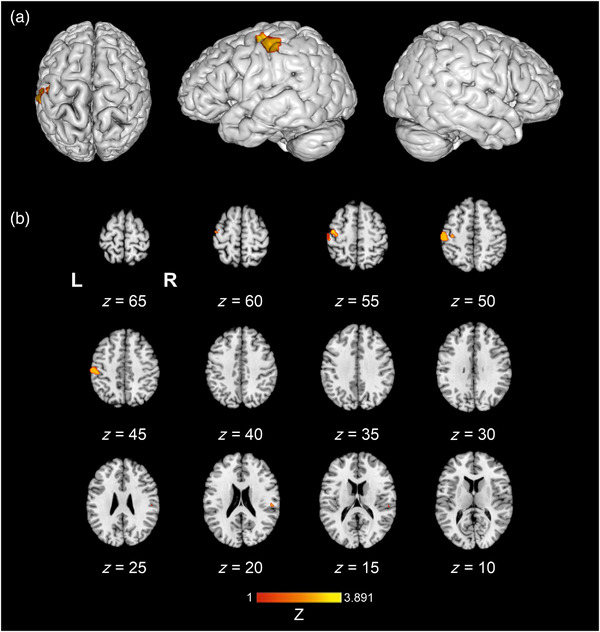
The location of significant clusters from contrast analysis of activation likelihood estimation (ALE) maps for a greater likelihood of texture perception > non‐haptic control relative to texture perception > haptic control. All clusters are overlaid onto a standardized Montreal Neurologic Institute (MNI) template anatomical brain in (a). Three‐dimensional (3D) surface projection from superior, left, and right views, respectively. (b) As a montage of coronal slices throughout the whole brain, L and R denote the left and right hemispheres, respectively. Relative *Z* scores are indicated by the color bar.

Contrast analysis comparing the ALE maps of concordant activations for each process pointed to a significantly greater likelihood of activation during general texture perception (texture perception > non‐haptic control) relative to texture‐specific perception (texture perception > haptic control) in three clusters (Figure 5, Table 5). Two left clusters included the SI and the SMG in the IPL, as well as the SI, MI, and premotor areas. The third cluster was located in the right IPL and corresponded to the SMG. The reverse contrast did not reveal any clusters indicative of increased activation likelihood estimates during the texture‐specific perception relative to general texture perception studies.

**TABLE 5:** Locations of significant clusters from contrast analysis of non‐haptic–haptic control.

Cluster #	Label	Volume (mm^3^)	BA	*x*	*y*	*z*	#Experiments	Extrema (*Z*)
1	Inferior parietal lobule L	1832	40	−53.3	−22.3	48.2	8	3.156
2	Postcentral gyrus L		3	−41.2	−12.3	55.2	4	3.719
	Postcentral gyrus L	640	3	−40.9	−18.7	54.5		3.156
3	Inferior parietal lobule R	232	40	55.4	−24.6	19.8	1	3.036

Abbreviations: BA, Brodmann area; L, left hemisphere; R, right hemisphere.

### 3.3 Sensitivity analysis

To assess the stability of results, 13 leave‐one‐out analyses (also known as jack‐knife analyses) were conducted, whereby the primary analysis of texture perception > control was rerun, each time excluding a different single study (Supporting Information 1; Radua & Mataix‐Cols, 2009; Radua et al., 2012). The sensitivity analysis confirmed the stability of the right SII cluster and left SI/IPL across all 13 studies. Leaving out Yang et al. (2017) resulted in the left PMv and left PI clusters no longer reaching significance. The SMA cluster was not identified when leaving out Mueller et al. (2019), Simões‐Franklin et al. (2011), Yang et al. (2017), and Kitada et al. (2006). In addition, leaving out Kitada et al. (2006) and Mueller et al. (2019) resulted in the loss of the right PMv cluster. The right PI cluster was not identified as significant when leaving out Kitada et al. (2006), Mueller et al. (2019), Sathian et al. (2011), and Stilla and Sathian (2008). Further, an additional cluster in the right PI/SII was identified when removing Simões‐Franklin et al. (2011), Mueller et al. (2019), Wang et al. (2016), Yang et al. (2021), and Kim et al. (2015). Removing Kitada et al. (2005) and Yang et al. (2021) led to the identification of a further cluster in the left dorsolateral prefrontal cortex (DLPFC). Finally, a cluster from the left SI to the left MI was uncovered when removing Kitada et al. (2005), Sathian et al. (2011), Simões‐Franklin et al. (2011), and Yang et al. (2017, 2021).

## 4 DISCUSSION

Findings from the primary ALE meta‐analysis of texture perception relative to control revealed nine significant clusters with activation in the left SI and SMG, bilateral PMv and SMA, and the right SII and PI, consistent with our hypotheses that texture perception would activate the sensorimotor regions that are well associated with tactile perception and movement planning and execution. The presence of haptic control conditions during texture perception identified activation in the left SII, in line with our hypothesis that texture processing activates brain regions associated with higher order processing. Further, contrast analyses revealed sensorimotor (SI, MI, and SMG) activations as more predominant in the non‐haptic control contrast than when controlling for other haptic processing, suggesting that texture‐specific processing may require the activation of higher order cortical regions.

Left SII was the only region selectively activated when contrasting texture processing with a haptic control, indicating its role in texture‐specific processing. The human parietal operculum comprises four distinct cytoarchitectonic areas (OP1−4; Eickhoff et al., 2006, 2007), with concordant activation in the left SII cluster corresponding to OP4. The SII has previously been implicated in higher order processes, including attention (Chen et al., 2008; Hämäläinen et al., 2000), learning (Mishkin, 1979; Ridley & Ettlinger, 1976), and roughness discrimination (Kitada et al., 2005; Sathian et al., 2011; Servos et al., 2001; Stilla & Sathian, 2008). Additionally, concordant activation was found in OP4 during a detect‐discriminate touch meta‐analysis (Morrison, 2016), and this region, along with OP1 and OP3, has been proposed as texture selective (Sathian et al., 2011; Stilla & Sathian, 2008). Research with non‐human primates shows that lesions to the SII result in deficits in texture and shape discrimination (Garcha & Ettlinger, 1980; Murray & Mishkin, 1984), while humans with lesions in the area suffer from tactile apraxia (Binkofski et al., 2001). (Jiang et al., 1997; Pruett et al., 2000; Sinclair & Burton, 1993). Furthermore, low‐ and high‐frequency vibrotactile cues elicit differential patterns of brain activation, with increased SII activation during high‐frequency vibrotactile stimulation (Chung et al., 2013; Francis et al., 2000; Han et al., 2013; Harrington & Hunter Downs, 2001; Ryun, Kim, Lee, et al., 2017). Concordant activation in the SII when contrasting with haptic control was observed only in response to textiles, which are more likely to induce high‐frequency vibrations due to their finer grained surface texture. Taken together the results of this meta‐analysis, it is likely that the SII plays a significant role in the higher order encoding of textural properties, which may be correlated with high‐frequency vibrotactile information.

Conjunction and contrast analyses were computed to examine similarities and differences between the processing of generic discriminative touch, by comparing texture processing to non‐haptic control conditions, relative to brain regions that are more selectively activated during texture processing/evaluations, which were determined by comparing texture processing to haptic control conditions. Concordant activation in the left SI (Brodmann's area; BA 3) and bilateral SMG was more likely to be activated during texture perception compared to non‐haptic control contrast, relative to touch minus haptic control contrast tasks that accounted for other aspects of discrimination (e.g., shape). Interestingly, the conjunction analysis did not identify any overlap of findings across studies with these differing approaches. Demonstrating that concordant activation of the SII cluster in the texture relative to haptic control ALE analysis may be specific to texture processing. These findings indicate that broad aspects of tactile information are processed in the sensorimotor areas, hence the dominance in contrasts which do not correct for haptic processing in the baseline. However, the absence of deeper SII or PI clusters indicates that important aspects of texture‐specific processing may occur in higher order medial regions such as the SII and insular cortex (Eck et al., 2016; Jiang et al., 1997; Roland et al., 1998), which may require careful consideration of experimental design, and particularly baseline, to investigate. This aligns with the presence of an SII cluster in the texture relative to haptic control ALE analysis.

Bilateral PI was found to be active when investigating texture processing compared to control (i.e., non‐haptic and haptic control conditions combined). The insular cortex has been linked with somatosensation (Kurth et al., 2010; Preusser et al., 2015) and has been associated with the intensity processing of thermosensory (Craig et al., 2000) and noxious stimuli (Frot et al., 2007; Iannetti et al., 2005). Roughness‐related activation has been reported in the parietal operculum and insula (Kitada et al., 2005). Therefore, the insula may play a role in the processing or evaluation of texture intensity. For the present meta‐analysis, 10 studies were included where participants were asked to complete an estimation or comparison task, evaluating textural features with a response (Gurtubay‐Antolin et al., 2018; Kitada et al., 2006; Mueller et al., 2019; Podrebarac et al., 2014; Sathian et al., 2011; Simões‐Franklin et al., 2011; Stilla & Sathian, 2008; Wang et al., 2016; Yang et al., 2017, 2021). The insula is anatomically interconnected to the prefrontal cortex (Burton & Sinclair, 2000; Preusser et al., 2015); as such, the insula is conceptualized as an integrative region associated with both sensory input (Craig et al., 2000; Jensen et al., 2016; Segerdahl et al., 2015) and cognitive processes, such as decision‐making (Gogolla, 2017; Uddin et al., 2017). Therefore, insula activation may reflect the integration of sensory input, which may be crucial for higher order cognitive decisions based on sensory features.

SI and SMG were found to be active when comparing texture processing to both control and non‐haptic control conditions. The SI processes tactile information and encompasses BA 3a, 3b, 1, and 2 (Case et al., 2016; Chapman, 1994; Lieber & Bensmaia, 2019, 2020; Lin et al., 1996; Purves et al., 2001). Concordant activation peaked in BA 2 of the left SI, which is associated with the processing of tactile and proprioceptive input (Hyvärinen & Poranen, 1978; Padberg et al., 2019; Pons et al., 1985). Furthermore, the tactile stimulation of the fingers is consistently associated with activation of BA 2 (Hlushchuk & Hari, 2006; Holmes et al., 2019; Janko et al., 2022; Puckett et al., 2020), suggesting concordant activation in the SI corresponds to the finger area. Active touch engages sensorimotor circuits in the PPC, including the SMG located in the IPL, and activation in these regions has been associated with sensorimotor integration (Arikan et al., 2021; Batista et al., 1999; Battaglia‐Mayer et al., 2000; Buneo et al., 2002; Buneo & Andersen, 2006; Ferraina et al., 1997; Hyvärinen, 1982; Lamp et al., 2019; Mountcastle et al., 1975; Naito et al., 2005; Snyder et al., 1997).

The MI demonstrated concordant activation only when compared to the non‐haptic control condition. Concordant activation of the MI, associated with the execution of voluntary movement (Kalaska & Rizzolatti, 2012), was observed to be predominantly engaged within the hand knob area (Bonzano et al., 2022; Hardwick et al., 2013; Puce et al., 1995; Yousry et al., 1997). An exploratory analysis of experiments employing active touch found concordant activation in premotor and motor areas, while passive touch did not (Supporting Information 2). However, two studies contributing to the concordant activation identified in the MI cluster employed dynamic passive touch (Kitada et al., 2005; Tang et al., 2021); therefore, concordance of MI activation may also be due to force exerted by the finger during passive paradigms rather than solely due to active touch (Dettmers et al., 1995).

Concordant activation was identified in both the bilateral PMv and SMA across two ALE analyses, one that considered all studies irrespective of the control condition and the other that only considered baselines that did not account for other haptic elements (i.e., non‐haptic control). The PMv is associated with both tactile processing (Avanzini et al., 2016; Ryun et al., 2023) and movement‐related functions, including grasping and object manipulation (Davare et al., 2006, 2008, 2009; Fogassi et al., 2001; Reader & Holmes, 2018; Vingerhoets et al., 2013). In non‐human primates, the PMv has been linked to the evaluation of sensory information for guiding motor action (Romo et al., 2004). Five studies that contributed to the bilateral PMv clusters involved active touch (Mueller et al., 2019; Sathian et al., 2011; Simões‐Franklin et al., 2011; Wang et al., 2016; Yang et al., 2021). Accordingly, SMA neurons discharge before and during coordinated voluntary movement (Tanji, 2001; Tanji & Shima, 1996), such as button pressing. Therefore, PMv and SMA activation may reflect the evaluation of sensory information to inform response behavior during experimental paradigms.

The current meta‐analysis is impacted by the limited number of studies, partially due to the absence of a standardized paradigm, resulting in the rejection of 130 studies due to stimuli/paradigm discrepancies. Therefore, establishing a standardized texture perception paradigm would benefit the field. Further, a vast range of tactile stimuli are used in texture perception paradigms, including gratings (Kitada et al., 2005, 2006), 3D‐printed textures (Mueller et al., 2019; Yang et al., 2021), dot patterns (Yang et al., 2017, 2021), and textiles (Gurtubay‐Antolin et al., 2018; Wang et al., 2016), each varying considerably in their tactile properties. Textiles are often finer grained and therefore are more likely to rely on vibrational cues generated through movement, while coarse textures such as gratings depend on distinct spatial patterns (Moungou et al., 2016; Weber et al., 2013). Consequently, findings are difficult to collate to investigate texture‐specific processing. In the future, a standardized battery of textural stimuli would aid researchers to align and compare findings across studies, laboratories, and geographical regions. Furthermore, concordant activation identified in the SI, MI, PMv, IPL, SII, and insula parallels human electrophysiological mapping studies (Ryun et al., 2023; Ryun, Kim, Jeon, et al., 2017; Ryun, Kim, Lee, et al., 2017), providing further evidence that these areas contribute to tactile perception. While a larger sample size of 17–20 studies is recommended to detect small effects (Eickhoff et al., 2016), the alignment of these findings suggests that the current findings are robust.

Studies identified by systematic review are limited by modest participant numbers. The recommended sample size for investigating sensorimotor effects with 3T scanners is a minimum of 20 participants, and preferably 27 participants (Thirion et al., 2007). The average number of participants recruited in the studies contributing to this meta‐analysis was 15 ± 4.63 (M ±  SD) with only three studies (Mueller et al., 2019; Yang et al., 2017, 2021) recruiting 20 participants or more. Therefore, contributing foci are potentially underpowered. However, a leave‐one‐out analysis was conducted to assess the sensitivity of results (Supporting Information 2; Acar et al., 2018), which demonstrated that clusters in the right SII and left SI/IPL were stable across all 13 studies. During leave‐one‐out analyses, additional clusters were identified in the left SII and left DLPFC and SI/MI, which may indicate that bilateral SII and higher order prefrontal regions are important for texture processing. However, the identification of concordant activation in these areas may be dependent on task design and/or stimuli utilized, hence the sensitivity to leave‐one‐out procedures. Thus, the importance of a standardized procedure in the field of texture processing is highlighted.

To conclude, findings revealed expected concordance in sensorimotor areas including higher order structures associated with top‐down mechanisms. Analysis of studies that included a haptic baseline to control for non‐textual processing revealed concordance solely in the left SII. Furthermore, the contrast analysis demonstrated that lateral SI and IPL are significantly more predominant when utilizing a resting baseline than in studies where textural aspects of discriminative touch are accounted for in the baseline. These findings point toward the preferential processing of texture in higher order structures, particularly the SII. Further research should carefully consider research design, and particularly the use of appropriate baseline contrasts to uncover the role of higher order structures in texture processing. Overall, the present study has furthered our understanding of texture perception, specifically when accounting for the influence of other haptic processes that offer unique insight into the neural correlates of texture‐related processing.

## AUTHOR CONTRIBUTIONS

**Jessica Henderson**: Conceptualization, methodology, formal analysis, investigation, data curation, writing—original draft, writing—review and editing, visualization, project administration. **Tyler Mari**: Investigation, writing—review and editing. **Danielle Hewitt**: Writing—review and editing. **Alice Newton-Fenner**: Writing—review and editing. **Timo Giesbrecht**: Conceptualization, funding acquisition, supervision. **Alan Marshall**: Conceptualization, supervision, writing—review and editing. **Andrej Stancák**: Conceptualization, supervision, writing—review and editing. **Nicholas Fallon**: Conceptualization, methodology, writing—review and editing, supervision, funding acquisition.

## CONFLICT OF INTEREST STATEMENT

The authors declare no conflicts of interest.

### PEER REVIEW

The peer review history for this article is available at https://publons.com/publon/10.1002/brb3.3264.

## Supporting information

Supplementary table S1. Locations of significant clusters when leaving out Gurtubay‐Antolin et al. (2018).
Supplementary table S2. Locations of significant clusters when leaving out Kim et al. (2015).
Supplementary table S3. Locations of significant clusters when leaving out Kitada et al. (2005).
Supplementary table S4. Locations of significant clusters when leaving out Kitada et al. (2006).
Supplementary table S5. Locations of significant clusters when leaving out Mueller et al. (2019).
Supplementary table S6. Locations of significant clusters when leaving out Podrebarac et al. (2014).
Supplementary table S7. Locations of significant clusters when leaving out Sathian et al. (2011).
Supplementary table S8. Locations of significant clusters when leaving out Simões‐Franklin et al. (2011).
Supplementary table S9. Locations of significant clusters when leaving out Stilla and Sathian (2008).
Supplementary table S10. Locations of significant clusters when leaving out Tang et al. (2021).
Supplementary table S11. Locations of significant clusters when leaving out Wang et al. (2016).
Supplementary table S12. Locations of significant clusters when leaving out Yang et al. (2017).
Supplementary table S13. Locations of significant clusters when leaving out Yang et al. (2021).

Supplementary table S14. Locations of significant clusters for both active and passive stimulation, with the exception of Simões‐Franklin et al. (2011).
Supplementary table S15.
Supplementary table S16.

## Data Availability

Data that support the findings of this study are openly available on Open Science Framework (Henderson et al., 2020).
