# Supplementary material for: The neural correlates of texture perception: A systematic review and activation likelihood estimation meta‐analysis of functional magnetic resonance imaging studies
Source: Brain Behav. 2023 Sep 25;13(11):e3264. doi: 10.1002/brb3.3264 (PMC10636420; doi:10.1002/brb3.3264)
Supplement: Supplementary file 1 — Supplementary table S1. Locations of significant clusters when leaving out Gurtubay‐Antolin et al. (2018). Supplementary table S2. Locations of significant clusters when leaving out Kim et al. (2015). Supplementary table S3. Locations of significant clusters when leaving out Kitada et al. (2005). Supplementary table S4. Locations of significant clusters when leaving out Kitada et al. (2006). Supplementary table S5. Locations of significant clusters when leaving out Mueller et al. (2019). Supplementary table S6. Locations of significant clusters when leaving out Podrebarac et al. (2014). Supplementary table S7. Locations of significant clusters when leaving out Sathian et al. (2011). Supplementary table S8. Locations of significant clusters when leaving out Simões‐Franklin et al. (2011). Supplementary table S9. Locations of significant clusters when leaving out Stilla and Sathian (2008). Supplementary table S10. Locations of significant clusters when leaving out Tang et al. (2021). Supplementary table S11. Locations of significant clusters when leaving out Wang et al. (2016). Supplementary table S12. Locations of significant clusters when leaving out Yang et al. (2017). Supplementary table S13. Locations of significant clusters when leaving out Yang et al. (2021). [file BRB3-13-e3264-s002.docx]

# Supplementary data file 1: Leave one out analysis

Thirteen “leave one out” analyses were conducted to assess the stability of the results. Below are the results from the primary analysis of texture perception > control each time excluding a different single study.

Gurtubay-Antolin et al. (2018)

Supplementary table 1. Locations of significant clusters when leaving out Gurtubay-Antolin et al. (2018).

| Cluster # | Label | Volume (mm^3^) | BA | x | y | z | # Experiments | ALE |
| --- | --- | --- | --- | --- | --- | --- | --- | --- |
| 1 | Insula R | 1760 | 13 | 58 | -20 | 20 | 7 | 0.021 |
|  | Postcentral Gyrus R |  | 3 | 64 | -16 | 22 |  | 0.021 |
| 2 | Postcentral Gyrus L | 1648 | 2 | -54 | -20 | 48 | 8 | 0.023 |
| 3 | Precentral Gyrus L | 1432 | 6 | -48 | 6 | 24 | 7 | 0.019 |
|  | Precentral Gyrus L |  | 6 | -58 | 8 | 28 |  | 0.014 |
|  | Precentral Gyrus L |  | 6 | -58 | 2 | 32 |  | 0.012 |
| 4 | Insula L | 1248 | 13 | -36 | -6 | 10 | 6 | 0.022 |
|  | Insula L |  | 13 | -42 | -4 | 2 |  | 0.014 |
| 5 | Inferior Frontal Gyrus R | 984 | 9 | 50 | 8 | 24 | 4 | 0.025 |
| 6 | Insula R | 832 | 13 | 40 | -8 | 8 | 5 | 0.020 |
| 7 | Superior Frontal Gyrus R | 832 | 6 | 4 | 16 | 48 | 4 | 0.018 |

BA, Brodmann Area; L, left hemisphere; R, right hemisphere.

Kim et al. (2015)

Supplementary table 2. Locations of significant clusters when leaving out Kim et al. (2015).

| Cluster # | Label | Volume (mm^3^) | BA | x | y | z | # Experiments | ALE |
| --- | --- | --- | --- | --- | --- | --- | --- | --- |
| 1 | Insula R | 1840 | 13 | 58 | -20 | 20 | 7 | 0.021 |
|  | Postcentral Gyrus R |  | 3 | 64 | -16 | 22 |  | 0.021 |
| 2 | Precentral Gyrus L | 1688 | 6 | -48 | 6 | 24 | 7 | 0.019 |
|  | Precentral Gyrus L |  | 6 | -58 | 8 | 28 |  | 0.014 |
|  | Precentral Gyrus L |  | 6 | -58 | 6 | 14 |  | 0.013 |
|  | Precentral Gyrus L |  | 6 | -58 | 2 | 32 |  | 0.012 |
| 3 | Inferior Parietal Lobule L | 1480 | 40 | -54 | -22 | 48 | 7 | 0.022 |
| 4 | Insula L | 1096 | 13 | -36 | -6 | 10 | 6 | 0.022 |
|  | Insula L |  | 13 | -42 | -4 | 0 |  | 0.013 |
| 5 | Inferior Frontal Gyrus R | 1048 | 9 | 50 | 8 | 24 | 4 | 0.025 |
| 6 | Insula R | 856 | 13 | 40 | -8 | 8 | 5 | 0.020 |
| 7 | Superior Frontal Gyrus R | 784 | 6 | 4 | 18 | 48 | 4 | 0.018 |
| 8 | Postcentral Gyrus L | 728 | 40 | -56 | -20 | 20 | 4 | 0.017 |

BA, Brodmann Area; L, left hemisphere; R, right hemisphere.

Kitada et al. (2005)

Supplementary table 3. Locations of significant clusters when leaving out Kitada et al. (2005).

| Cluster # | Label | Volume (mm^3^) | BA | x | y | z | # Experiments | ALE |
| --- | --- | --- | --- | --- | --- | --- | --- | --- |
| 1 | Precentral Gyrus L | 1672 | 6 | -48 | 6 | 24 | 7 | 0.019 |
|  | Precentral Gyrus L |  | 6 | -58 | 8 | 28 |  | 0.014 |
|  | Precentral Gyrus L |  | 6 | -58 | 6 | 14 |  | 0.013 |
|  | Precentral Gyrus L |  | 6 | -58 | 2 | 32 |  | 0.012 |
| 2 | Postcentral Gyrus L | 1488 | 2 | -54 | -20 | 50 | 7 | 0.022 |
| 3 | Postcentral Gyrus R | 1376 | 3 | 64 | -16 | 22 | 6 | 0.02 |
|  | Insula R |  | 13 | 54 | -20 | 20 |  | 0.016 |
| 4 | Insula L | 1328 | 13 | -36 | -6 | 10 | 6 | 0.022 |
|  | Insula L |  | 13 | -42 | -4 | 2 |  | 0.014 |
| 5 | Inferior Frontal Gyrus R | 1032 | 9 | 50 | 8 | 24 | 4 | 0.025 |
| 6 | Insula R | 856 | 13 | 40 | -8 | 8 | 5 | 0.02 |
| 7 | Superior Frontal Gyrus R | 784 | 6 | 4 | 18 | 48 | 4 | 0.018 |
| 8 | Middle Frontal Gyrus L | 656 | 46 | -42 | 40 | 12 | 3 | 0.019 |

BA, Brodmann Area; L, left hemisphere; R, right hemisphere.

Kitada et al. (2006)

Supplementary table 4. Locations of significant clusters when leaving out Kitada et al. (2006).

| Cluster # | Label | Volume (mm^3^) | BA | x | y | z | # Experiments | ALE |
| --- | --- | --- | --- | --- | --- | --- | --- | --- |
| 1 | Inferior Frontal Gyrus L | 1512 | 9 | -50 | 6 | 24 | 6 | 0.017 |
|  | Inferior Frontal Gyrus L |  | 9 | -56 | 6 | 22 |  | 0.016 |
|  | Precentral Gyrus L |  | 6 | -58 | 8 | 28 |  | 0.014 |
|  | Precentral Gyrus L |  | 6 | -58 | 6 | 14 |  | 0.013 |
|  | Precentral Gyrus L |  | 6 | -58 | 2 | 32 |  | 0.012 |
| 2 | Postcentral Gyrus R | 1392 | 3 | 64 | -16 | 22 | 6 | 0.021 |
| 3 | Postcentral Gyrus L | 1328 | 2 | -54 | -20 | 48 | 7 | 0.021 |
| 4 | Insula L | 1168 | 13 | -36 | -6 | 10 | 5 | 0.022 |
| 5 | Postcentral Gyrus L | 768 | 3 | -44 | -12 | 58 | 4 | 0.016 |
|  | Precentral Gyrus L |  | 4 | -38 | -20 | 52 |  | 0.013 |

BA, Brodmann Area; L, left hemisphere; R, right hemisphere.

Mueller et al. (2019)

Supplementary table 5. Locations of significant clusters when leaving out Mueller et al. (2019).

| Cluster # | Label | Volume (mm^3^) | BA | x | y | z | # Experiments | ALE |
| --- | --- | --- | --- | --- | --- | --- | --- | --- |
| 1 | Insula R | 1528 | 13 | 58 | -20 | 20 | 5 | 0.021 |
| 2 | Postcentral Gyrus L | 1400 | 1 | -58 | -22 | 46 | 7 | 0.019 |
|  | Postcentral Gyrus L |  | 40 | -50 | -28 | 56 |  | 0.010 |
| 3 | Insula L | 1096 | 13 | -38 | -8 | 10 | 5 | 0.016 |
|  | Insula L |  | 13 | -42 | -4 | 2 |  | 0.014 |
| 4 | Precentral Gyrus L | 888 | 6 | -48 | 6 | 24 | 4 | 0.019 |
| 5 | Postcentral Gyrus L | 832 | 40 | -56 | -20 | 20 | 4 | 0.017 |

BA, Brodmann Area;L, left hemisphere; R, right hemisphere.

Podrebarac et al. (2014)

Supplementary table 6. Locations of significant clusters when leaving out Podrebarac et al. (2014).

| Cluster # | Label | Volume (mm^3^) | BA | x | y | z | # Experiments | ALE |
| --- | --- | --- | --- | --- | --- | --- | --- | --- |
| 1 | Insula R | 1760 | 13 | 58 | -20 | 20 | 7 | 0.021 |
|  | Postcentral Gyrus R |  | 3 | 64 | -16 | 22 |  | 0.021 |
| 2 | Postcentral Gyrus L | 1648 | 2 | -54 | -20 | 48 | 8 | 0.023 |
| 3 | Precentral Gyrus L | 1432 | 6 | -48 | 6 | 24 | 7 | 0.019 |
|  | Precentral Gyrus L |  | 6 | -58 | 8 | 28 |  | 0.014 |
|  | Precentral Gyrus L |  | 6 | -58 | 2 | 32 |  | 0.012 |
| 4 | Insula L | 1240 | 13 | -36 | -6 | 10 | 6 | 0.022 |
|  | Insula L |  | 13 | -42 | -4 | 2 |  | 0.014 |
| 5 | Inferior Frontal Gyrus R | 984 | 9 | 50 | 8 | 24 | 4 | 0.025 |
| 6 | Superior Frontal Gyrus R | 832 | 6 | 4 | 16 | 48 | 4 | 0.018 |
| 7 | Insula R | 824 | 13 | 40 | -8 | 8 | 5 | 0.020 |

BA, Brodmann Area; L, left hemisphere; R, right hemisphere.

Sathian et al. (2011)

Supplementary table 7. Locations of significant clusters when leaving out Sathian et al. (2011).

| Cluster # | Label | Volume (mm^3^) | BA | x | y | z | # Experiments | ALE |
| --- | --- | --- | --- | --- | --- | --- | --- | --- |
| 1 | Insula R | 1792 | 13 | 58 | -20 | 20 | 7 | 0.021 |
|  | Postcentral Gyrus R |  | 3 | 64 | -16 | 22 |  | 0.021 |
| 2 | Postcentral Gyrus L | 1704 | 2 | -54 | -20 | 48 | 8 | 0.023 |
| 3 | Inferior Frontal Gyrus L | 1056 | 9 | -58 | 6 | 22 | 5 | 0.016 |
|  | Precentral Gyrus L |  | 6 | -58 | 8 | 28 |  | 0.014 |
|  | Precentral Gyrus L |  | 6 | -58 | 2 | 32 |  | 0.012 |
| 4 | Inferior Frontal Gyrus R | 1008 | 9 | 50 | 8 | 24 | 4 | 0.025 |
| 5 | Superior Frontal Gyrus R | 864 | 6 | 4 | 16 | 48 | 4 | 0.018 |
| 6 | Insula L | 760 | 13 | -38 | -6 | 8 | 4 | 0.015 |
|  | Insula L |  | 13 | -42 | -4 | 2 |  | 0.014 |
| 7 | Postcentral Gyrus L | 656 | 3 | -44 | -12 | 58 | 4 | 0.016 |
|  | Precentral Gyrus L |  | 4 | -38 | -20 | 52 |  | 0.013 |
| 8 | Postcentral Gyrus L | 632 | 40 | -56 | -20 | 20 | 4 | 0.017 |

BA, Brodmann Area; L, left hemisphere; R, right hemisphere.

Simões-Franklin et al. (2011)

Supplementary table 8. Locations of significant clusters when leaving out Simões-Franklin et al. (2011).

| Cluster # | Label | Volume (mm^3^) | BA | x | y | z | # Experiments | ALE |
| --- | --- | --- | --- | --- | --- | --- | --- | --- |
| 1 | Postcentral Gyrus L | 1560 | 2 | -54 | -20 | 48 | 6 | 0.023 |
| 2 | Precentral Gyrus L | 1360 | 6 | -48 | 6 | 24 | 6 | 0.019 |
|  | Precentral Gyrus L |  | 6 | -58 | 6 | 14 |  | 0.013 |
| 3 | Insula R | 1328 | 13 | 54 | -22 | 20 | 6 | 0.018 |
|  | Inferior Parietal Lobule R |  | 40 | 64 | -16 | 24 |  | 0.017 |
| 4 | Inferior Frontal Gyrus R | 992 | 9 | 50 | 8 | 24 | 4 | 0.025 |
| 5 | Insula R | 872 | 13 | 40 | -8 | 8 | 5 | 0.020 |
| 6 | Insula L | 864 | 13 | -36 | -6 | 10 | 4 | 0.021 |
| 7 | Postcentral Gyrus L | 760 | 40 | -56 | -20 | 20 | 4 | 0.017 |
| 8 | Postcentral Gyrus L | 744 | 3 | -44 | -12 | 58 | 4 | 0.016 |
|  | Precentral Gyrus L |  | 4 | -38 | -20 | 52 |  | 0.013 |

BA, Brodmann Area; L, left hemisphere; R, right hemisphere.

Stilla and Sathian (2008)

Supplementary table 9. Locations of significant clusters when leaving out Stilla and Sathian (2008).

| Cluster # | Label | Volume (mm^3^) | BA | x | y | z | # Experiments | ALE |
| --- | --- | --- | --- | --- | --- | --- | --- | --- |
| 1 | Insula R | 1704 | 13 | 58 | -20 | 20 | 7 | 0.021 |
|  | Postcentral Gyrus R |  | 3 | 64 | -16 | 22 |  | 0.021 |
| 2 | Postcentral Gyrus L | 1640 | 2 | -54 | -20 | 48 | 8 | 0.023 |
| 3 | Precentral Gyrus L | 1424 | 6 | -48 | 6 | 24 | 7 | 0.019 |
|  | Precentral Gyrus L |  | 6 | -58 | 8 | 28 |  | 0.014 |
|  | Precentral Gyrus L |  | 6 | -58 | 2 | 32 |  | 0.012 |
| 4 | Inferior Frontal Gyrus R | 984 | 9 | 50 | 8 | 24 | 4 | 0.025 |
| 5 | Insula L | 960 | 13 | -38 | -6 | 10 | 5 | 0.019 |
|  | Insula L |  | 13 | -42 | -4 | 2 |  | 0.014 |
| 6 | Superior Frontal Gyrus R | 832 | 6 | 4 | 16 | 48 | 4 | 0.018 |

BA, Brodmann Area; L, left hemisphere; R, right hemisphere.

Tang et al. (2021)

Supplementary table 10. Locations of significant clusters when leaving out Tang et al. (2021).

| Cluster # | Label | Volume (mm^3^) | BA | x | y | z | # Experiments | ALE |
| --- | --- | --- | --- | --- | --- | --- | --- | --- |
| 1 | Precentral Gyrus L | 1440 | 6 | -48 | 6 | 24 | 7 | 0.019 |
|  | Precentral Gyrus L |  | 6 | -58 | 8 | 28 |  | 0.014 |
|  | Precentral Gyrus L |  | 6 | -58 | 2 | 32 |  | 0.012 |
| 2 | Insula R | 1400 | 13 | 58 | -20 | 20 | 5 | 0.021 |
| 3 | Insula L | 1248 | 13 | -36 | -6 | 10 | 6 | 0.022 |
|  | Insula L |  | 13 | -42 | -4 | 2 |  | 0.014 |
| 4 | Inferior Parietal Lobule L | 1224 | 40 | -54 | -22 | 48 | 6 | 0.019 |
| 5 | Inferior Frontal Gyrus R | 984 | 9 | 50 | 8 | 24 | 4 | 0.025 |
| 6 | Insula R | 840 | 13 | 40 | -8 | 8 | 5 | 0.020 |
| 7 | Superior Frontal Gyrus R | 840 | 6 | 4 | 16 | 48 | 4 | 0.018 |

BA, Brodmann Area; L, left hemisphere; R, right hemisphere.

Wang et al. (2016)

Supplementary table 11. Locations of significant clusters when leaving out Wang et al. (2016).

| Cluster # | Label | Volume (mm^3^) | BA | x | y | z | # Experiments | ALE |
| --- | --- | --- | --- | --- | --- | --- | --- | --- |
| 1 | Insula R | 1408 | 13 | 58 | -20 | 20 | 6 | 0.021 |
| 2 | Insula L | 1272 | 13 | -36 | -6 | 10 | 6 | 0.022 |
|  | Insula L |  | 13 | -42 | -4 | 2 |  | 0.014 |
| 3 | Postcentral Gyrus L | 1248 | 2 | -54 | -20 | 48 | 6 | 0.023 |
| 4 | Precentral Gyrus L | 1040 | 6 | -48 | 6 | 24 | 5 | 0.019 |
|  | Precentral Gyrus L |  | 6 | -58 | 8 | 30 |  | 0.013 |
|  | Precentral Gyrus L |  | 6 | -58 | 2 | 32 |  | 0.012 |
| 5 | Inferior Frontal Gyrus R | 968 | 9 | 50 | 8 | 24 | 4 | 0.025 |
| 6 | Superior Frontal Gyrus R | 864 | 6 | 4 | 16 | 48 | 4 | 0.018 |
| 7 | Insula R | 848 | 13 | 40 | -8 | 8 | 5 | 0.020 |
| 8 | Postcentral Gyrus L | 664 | 40 | -56 | -20 | 20 | 4 | 0.017 |

BA, Brodmann Area; L, left hemisphere; R, right hemisphere.

Yang et al. (2017)

Supplementary table 12. Locations of significant clusters when leaving out Yang et al. (2017).

| Cluster # | Label | Volume (mm^3^) | BA | x | y | z | # Experiments | ALE |
| --- | --- | --- | --- | --- | --- | --- | --- | --- |
| 1 | Postcentral Gyrus R | 1440 | 3 | 64 | -16 | 22 | 6 | 0.020 |
| 2 | Postcentral Gyrus L | 1400 | 2 | -54 | -20 | 50 | 6 | 0.021 |
| 3 | Insula R | 992 | 13 | 40 | -8 | 8 | 5 | 0.020 |
| 4 | Postcentral Gyrus L | 872 | 3 | -44 | -12 | 58 | 4 | 0.016 |
|  | Precentral Gyrus L |  | 4 | -38 | -20 | 52 |  | 0.013 |
| 5 | Inferior Frontal Gyrus R | 856 | 9 | 48 | 8 | 26 | 3 | 0.018 |
|  | Inferior Frontal Gyrus R |  | 9 | 62 | 10 | 22 |  | 0.011 |

BA, Brodmann Area; L, left hemisphere; R, right hemisphere.

Yang et al. (2021)

Supplementary table 13. Locations of significant clusters when leaving out Yang et al. (2021).

| Cluster # | Label | Volume (mm^3^) | BA | x | y | z | # Experiments | ALE |
| --- | --- | --- | --- | --- | --- | --- | --- | --- |
| 1 | Insula R | 1784 | 13 | 58 | -20 | 20 | 7 | 0.021 |
|  | Postcentral Gyrus R |  | 3 | 64 | -16 | 22 |  | 0.021 |
| 2 | Postcentral Gyrus L | 1736 | 2 | -54 | -20 | 48 | 8 | 0.023 |
| 3 | Insula L | 1144 | 13 | -36 | -6 | 10 | 5 | 0.021 |
|  | Insula L |  | 13 | -42 | -4 | 2 |  | 0.014 |
| 4 | Superior Frontal Gyrus R | 888 | 6 | 4 | 16 | 48 | 4 | 0.018 |
| 5 | Inferior Frontal Gyrus L | 840 | 9 | -58 | 6 | 22 | 4 | 0.016 |
|  | Precentral Gyrus L |  | 6 | -58 | 8 | 30 |  | 0.013 |
|  | Precentral Gyrus L |  | 6 | -58 | 2 | 32 |  | 0.012 |
|  | Precentral Gyrus L |  | 6 | -46 | 6 | 26 |  | 0.011 |
| 6 | Inferior Frontal Gyrus R | 696 | 9 | 50 | 8 | 24 | 3 | 0.019 |
| 7 | Postcentral Gyrus L | 680 | 40 | -56 | -20 | 20 | 4 | 0.017 |
| 8 | Insula R | 664 | 13 | 40 | -8 | 8 | 4 | 0.019 |
| 9 | Postcentral Gyrus L | 656 | 3 | -44 | -12 | 58 | 4 | 0.016 |
|  | Precentral Gyrus L |  | 4 | -38 | -20 | 52 |  | 0.013 |
| 10 | Middle Frontal Gyrus L | 624 | 46 | -42 | 40 | 12 | 3 | 0.019 |

BA, Brodmann Area; L, left hemisphere; R, right hemisphere.
