# Supplementary material for: The neural correlates of texture perception: A systematic review and activation likelihood estimation meta‐analysis of functional magnetic resonance imaging studies
Source: Brain Behav. 2023 Sep 25;13(11):e3264. doi: 10.1002/brb3.3264 (PMC10636420; doi:10.1002/brb3.3264)
Supplement: Supplementary file 2 — Supplementary table S14. Locations of significant clusters for both active and passive stimulation, with the exception of Simões‐Franklin et al. (2011). Supplementary table S15. Supplementary table S16. [file BRB3-13-e3264-s001.docx]

# Supplementary data file 2: Active and Passive stimulation

An exploratory analysis was conducted to assess the differences between studies delivering tactile stimulation either through passive or active touch. Below are the results from the primary analysis of texture perception > control each, with the exception of Simões-Franklin et al. (2011) as the coordinates extracted were from activation via both active and passive touch combined.

## Primary analysis

### Pooled analysis

Supplementary table 14. Locations of significant clusters for both active and passive stimulation, with the exception of Simões-Franklin et al. (2011).

| Cluster # | Label | Volume(mm^3^) | BA | x | y | z | # Experiments | ALE |
| --- | --- | --- | --- | --- | --- | --- | --- | --- |
| 1 | Postcentral Gyrus L | 1560 | 2 | -54 | -20 | 48 | 6 | 0.023 |
| 2 | Precentral Gyrus L | 1360 | 6 | -48 | 6 | 24 | 6 | 0.019 |
|  | Precentral Gyrus L |  | 6 | -58 | 6 | 14 |  | 0.013 |
| 3 | Insula R | 1328 | 13 | 54 | -22 | 20 | 6 | 0.018 |
|  | Inferior Parietal Lobule R |  | 40 | 64 | -16 | 24 |  | 0.017 |
| 4 | Inferior Frontal Gyrus R | 992 | 9 | 50 | 8 | 24 | 4 | 0.025 |
| 5 | Insula R | 872 | 13 | 40 | -8 | 8 | 5 | 0.020 |
| 6 | Insula L | 864 | 13 | -36 | -6 | 10 | 4 | 0.021 |
| 7 | Postcentral Gyrus L | 760 | 40 | -56 | -20 | 20 | 4 | 0.017 |
| 8 | Postcentral Gyrus L | 744 | 3 | -44 | -12 | 58 | 4 | 0.016 |
|  | Precentral Gyrus L |  | 4 | -38 | -20 | 52 |  | 0.013 |

BA, Brodmann Area; L, left hemisphere; R, right hemisphere.

### Active touch

Supplementary table 15

| Cluster # | Label | Volume(mm^3^) | BA | x | y | z | # Experiments | ALE |
| --- | --- | --- | --- | --- | --- | --- | --- | --- |
| 1 | Insula L | 936 | 13 | -36 | -6 | 12 | 4 | 0.017 |
| 2 | Insula R | 920 | 13 | 40 | -6 | 10 | 4 | 0.016 |
| 3 | Precentral Gyrus L | 832 | 6 | -48 | 6 | 24 | 4 | 0.015 |
|  | Precentral Gyrus L |  | 6 | -60 | 6 | 22 |  | 0.012 |
| 4 | Postcentral Gyrus L | 792 | 3 | -42 | -12 | 56 | 3 | 0.011 |
|  | Precentral Gyrus L |  | 4 | -30 | -20 | 62 |  | 0.011 |
|  | Precentral Gyrus L |  | 4 | -34 | -18 | 60 |  | 0.010 |
|  | Precentral Gyrus L |  | 4 | -36 | -18 | 56 |  | 0.009 |
|  | Precentral Gyrus L |  | 4 | -36 | -14 | 64 |  | 0.008 |

BA, Brodmann Area; L, left hemisphere; R, right hemisphere.

### Passive touch

Supplementary table 16

| Cluster # | Label | Volume(mm^3^) | BA | x | y | z | # Experiments | ALE |
| --- | --- | --- | --- | --- | --- | --- | --- | --- |
| 1 | Postcentral Gyrus L | 992 | 1 | -58 | -22 | 46 | 4 | 0.018 |
|  | Postcentral Gyrus L |  | 40 | -50 | -28 | 58 |  | 0.009 |
| 2 | Insula R | 936 | 13 | 56 | -22 | 20 | 3 | 0.018 |
| 3 | Postcentral Gyrus L | 664 | 40 | -56 | -22 | 20 | 3 | 0.014 |

BA, Brodmann Area; L, left hemisphere; R, right hemisphere.

## Secondary analysis

The contrast and conjunction analysis comparing the ALE maps of concordant activations for active and passive touch types did not reveal any significant differences between the two types of stimulation.
